# Return to work after periacetabular osteotomy: workload as a determinant of occupational trajectories

**DOI:** 10.1302/2633-1462.76.BJO-2026-0047.R1

**Published:** 2026-06-01

**Authors:** Vincent J. Leopold, Sophia Steinhaus, Benita Dübber, Luis A. Becker, Carsten Perka

**Affiliations:** 1 Charité – University Medicine Berlin, Center for Musculoskeletal Surgery, Berlin, Germany; 2 Charité – University Medicine Berlin, Berlin, Germany

**Keywords:** Periacetabular osteotomy, Return to work, Workload, Outcomes, Midterm, Hip dysplasia, Patient reported outcomes, periacetabular osteotomy, Hip, Numeric Rating Scale, logistic regression analysis, Hip disability and Osteoarthritis Outcome Score, preoperative pain, Patient-acceptable symptom state (PASS), Developmental dysplasia of the hip (DDH), total hip arthroplasty, osteoarthritis

## Abstract

**Aims:**

Occupational outcomes after periacetabular osteotomy (PAO) remain underexplored. The aims were to: 1) to determine rate and duration to return to work (RTW) after PAO; 2) to analyze frequency and reasons for occupational change; and 3) assess career trajectories of students undergoing PAO.

**Methods:**

We retrospectively analyzed 117 patients who underwent PAO between January 2015 and June 2017, with a mean follow-up of 63.2 months (SD 10.2). A total of 90 patients were employed; 27 were students. Work-related outcomes were assessed via questionnaire, including RTW status, duration of work absence, occupational change, and whether PAO was considered reason for job change. Preoperative workload was assessed by the Reich Committee for Working Time Determination (REFA) classification. Returnees and non-returnees were compared, and predictors of occupational change were assessed with logistic regression.

**Results:**

Among 90 employed patients, 58 (64.4%) returned to their preoperative occupation, while 32 (35.6%) changed jobs. Overall, 14 patients (15.6% of all employed patients) reported that the hip that had undergone PAO was the reason for occupational change. Non-returnees reported higher preoperative pain (Numeric Rating Scale for pain 7.9 vs 7.0, p = 0.027), worse Hip disability and Osteoarthritis Outcome Score (HOOS)-Symptoms (42.3 vs 55.6, p = 0.038) and HOOS-Activities in daily living (44.1 vs 60.5, p = 0.015), higher workload-grades (0 (0 to 1) vs 2 (1 to 3), p < 0.001), and longer work absence (33.4 vs 26.7 weeks, p = 0.024). Logistic regression identified workload as the only independent predictor of occupational change (odds ratio up to 13.1 for heavier workload). The median time to job change was 86 weeks (IQR 10 to 208). Most changes occurred between one and two years postoperatively. Among students, 87.5% were employed at follow-up, most in sedentary (50%) or light (43%) occupations. Only one reported that PAO had influenced career choice.

**Conclusion:**

PAO enables the majority of young adults to RTW, but more than one third adapt their occupation in the mid-term, with higher preoperative workload being the key determinant. Despite improved hip function, occupational adaptations remained common, while long-term career trajectories in students were largely unaffected.

Cite this article: *Bone Jt Open* 2026;7(6):704–712.

## Introduction

Developmental dysplasia of the hip (DDH) is a recognized cause of premature osteoarthritis, and may lead to pain, functional impairment, and reduced activity levels in adolescents and young adults.^1-4^ Periacetabular osteotomy (PAO) has become the standard joint-preserving treatment for symptomatic DDH in the symptomatic pre-arthritic hip joint, with favourable mid- to long-term results regarding hip function and quality of life (QoL), as well as preservation of the native hip joint.^2,5-10^ While return to sports after PAO has been studied extensively, evidence on return to work (RTW) remains scarce.^11-14^

Due to the nature of the indication, the mostly young patients undergoing PAO are typically in the early phase of their working life. At the time of surgery, most are either already employed or in a stage of vocational training that qualifies them for subsequent employment. In this context, the ability to resume or initiate occupational activity after PAO represents a key functional and socioeconomic outcome. Despite its importance, mid- to long-term data on occupational outcomes following PAO are limited.

The aims of this investigation were therefore to: 1) determine the rate and duration of RTW following PAO; 2) analyze the frequency and nature of occupational changes after surgery; and 3) describe the professional trajectories of patients who were students at the time of surgery, including a possible influence of PAO on career choice.

We hypothesized that: 1) most patients would RTW within one year after PAO; 2) patients with physically demanding preoperative occupations would be more likely to change jobs after surgery; and 3) PAO would not influence long-term career choice in patients who underwent surgery during their training.

## Methods

### Study design

We performed a retrospective analysis of prospectively collected data from the institutional PAO database of a single university hospital. Approval from the local ethics committee was obtained prior to study initiation (EA1/052/21). The study was conducted in accordance with the Strengthening the Reporting of Observational Studies in Epidemiology (STROBE) guidelines. During the inclusion period between January 2015 and June 2017, a total of 202 PAOs were performed, of whom 117 fulfilled the inclusion criteria and were included in the present analysis. Of these cases, 90 were working at the time of PAO and 27 were students at the time of PAO. Of the working patients, 58 returned to work after PAO (returnees) and 32 did not return to their preoperative occupation (non-returnees).

### Patient selection

**Fig. 1:**
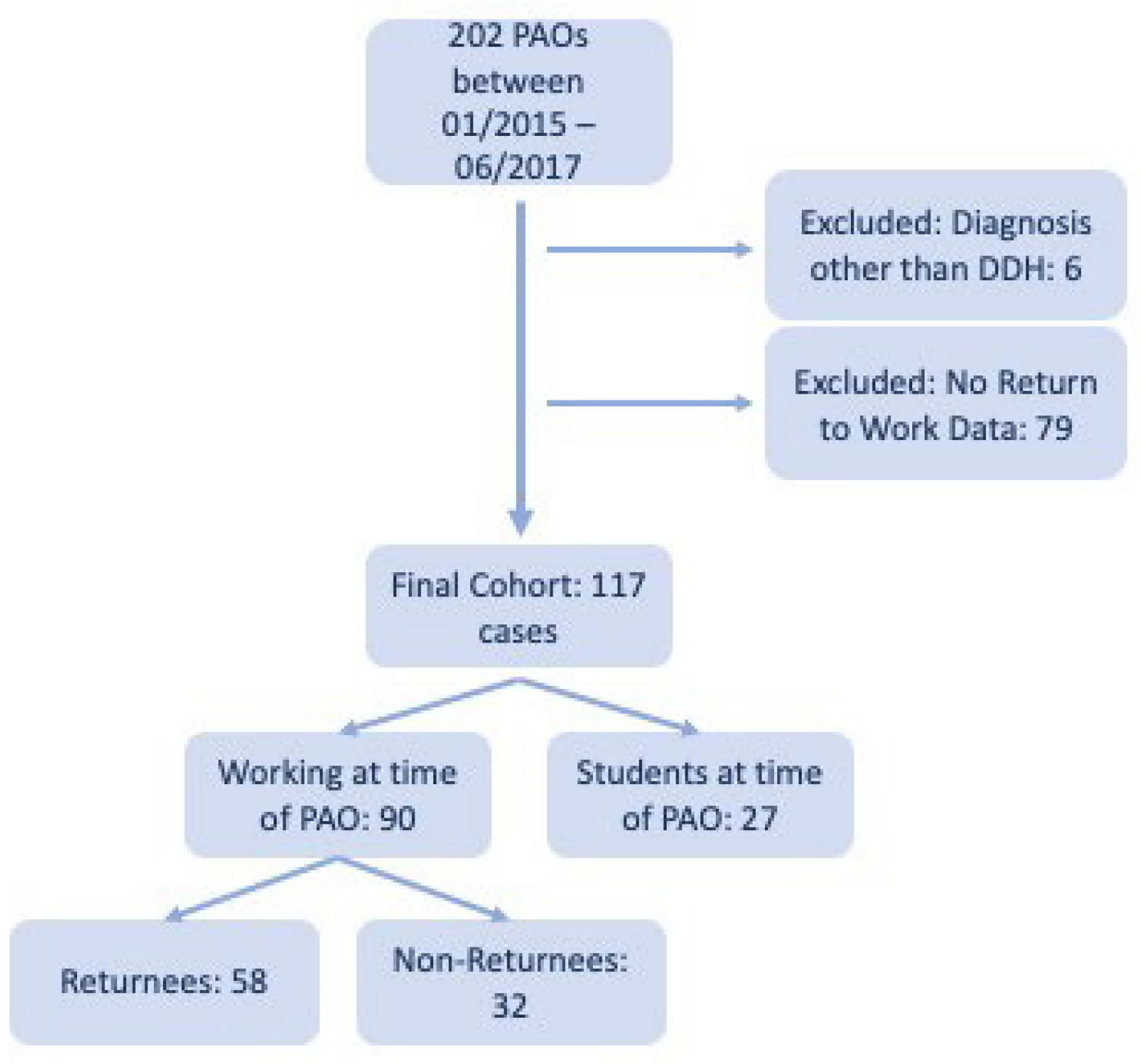
CONSORT flowchart of case selection. DDH, developmental dysplasia of the hip; PAO, periacetabular osteotomy.

Inclusion criteria were a primary diagnosis of symptomatic DDH confirmed on standardized anteroposterior pelvic radiographs, complete preoperative and postoperative clinical data, and completed return-to-work questionnaires. Exclusion criteria were PAO performed for other diagnoses such as acetabular retroversion, radiological signs of osteoarthritis beyond Tönnis grade 1, incomplete datasets, or missing informed consent. All patients included in the working cohort were employed at the time of surgery; patients without employment were not part of the return-to-work analysis. Students were analyzed separately as described. A detailed illustration of case selection is shown in Figure 1.

### Participant characteristics

A total of 117 patients were included in the analysis. The mean age at the time of surgery was 28.3 years (SD 7.5, 15 to 45), and the mean BMI was 24.4 kg/m² (SD 4.7, 17.0 to 35.9).

### Data collection

Pre- and postoperative data were obtained from a prospectively maintained institutional database. All patients completed standardized questionnaires at baseline (preoperatively) and at the most recent follow-up. The mean follow-up duration was 63.2 months (SD 10.2). The following validated hip-specific patient-reported outcome measures (PROMs) were collected: International Hip Outcome Tool (iHOT-12);^15^ Subjective Hip Value (SHV);^16^ Hip disability and Osteoarthritis Outcome Score (HOOS),^17^ including all sub-scales (Symptoms, Pain, Activities in daily living (ADL), QoL); and pain assessed on a Numeric Rating Scale (NRS, 0 to 10).^18^ Patient-acceptable symptom state (PASS) was assessed using the iHOT-12 at final follow-up. PASS achievement was defined according to previously established and widely accepted iHOT-12 thresholds (> 65), reflecting the postoperative score beyond which patients consider their symptom state satisfactory.^19^ Conversion to total hip arthroplasty was also assessed.

### Work-related outcomes

Work-related outcomes were assessed using a structured return-to-work questionnaire. RTW was defined as resumption of any occupational activity after PAO. Returnees were defined as patients who resumed and remained in their preoperative occupation at final follow-up. Non-returnees were defined as patients who were not working in their preoperative occupation at final follow-up, irrespective of whether they had initially returned to that occupation after surgery. The questionnaire included information on return-to-work status (yes/no), the duration of work absence in weeks, and whether the PAO was considered the reason for an occupational change. In cases where PAO was reported as the reason, patients were asked to provide additional free-text explanations. Furthermore, patients were asked to specify the timepoint of occupational change in weeks after surgery. For patients who were students at the time of surgery, follow-up information on occupational status and the influence of PAO on career choice was obtained via additional structured telephone interviews.

### Workload classification

Pre- and postoperative physical workload was classified according to the Reich Committee for Working Time Determination (REFA) classification system,^14^ which defines the type of work according to work duties and workload. This validated classification system has previously been applied in orthopaedic surgery to report rates of RTW.^14^ A description of workload according to the REFA classification is shown in Table I. The REFA classification was assigned retrospectively by the investigators based on detailed patient-reported job descriptions using standardized REFA criteria.

**Table I.** Reich Committee for Working Time Determination classification of physical workload with examples of work tasks.

Workload	REFA classification	Description
Sedentary work	Grade 0	Work without any special physical strain, like deskwork
Light manual work	Grade 1	Handling light work, lengthy standing, or walking
Heavy manual work	Grade 2	Handling a 1 kg to 3 kg control device, carrying loads of 10 kg to 15 kg, climbing stairs or ladders without a load
	Grade 3	Carrying loads of 20 kg to 30 kg, climbing stairs or ladders with a moderate load, moderate work in a tense work posture
	Grade 4	Carrying loads of more than 50 kg, climbing with a heavy load, hard work in a tense work posture

REFA, Reich Committee for Working Time Determination.

### Outcome measures

The primary endpoints of this study were RTW, changes in occupation and workload according to the REFA classification, and the potential influence of PAO on career choice in the sub-group of students. Secondary endpoints included changes in PROMs from pre- to postoperative follow-up and a descriptive analysis of occupational outcomes in the student sub-group. Radiological parameters (lateral centre-edge angle, Tönnis angle, anterior and posterior wall index, femoral head extrusion index) were measured on standardized anteroposterior pelvic radiographs and analyzed descriptively.

### Statistical analysis

Continuous variables were reported as mean, SD, and range, and categorical variables as absolute numbers and percentages. The distribution of continuous data was examined using the Shapiro-Wilk test. For normally distributed data, the *t*-test was applied, and the chi-squared test was used for categorical data. For non-normally distributed data, the Mann-Whitney U test was used for independent samples and the Wilcoxon signed-rank test for paired comparisons. Logistic regression analysis was performed to identify predictors of occupational change. To identify independent predictors for occupational change after PAO, a binary logistic regression analysis was performed with ‘job change’ (yes/no) as the dependent variable. Seven preselected variables were included in the model: preoperative pain level (NRS), functional status (iHOT-12, HOOS Symptoms, and HOOS ADL), their respective pre-post differences, and preoperative workload classification according to the REFA system. In a predefined sensitivity analysis, the outcome was restricted to hip-related occupational change, defined as job changes for which patients explicitly reported PAO as the reason. Given the limited number of hip-related events, this analysis was performed using a parsimonious univariable logistic regression model with preoperative workload (REFA classification) as the primary predictor. To assess potential selection bias related to missing preoperative job data, we compared baseline demographics (age, BMI, and sex distribution) between patients with available preoperative job information and those without. A p-value < 0.05 was considered statistically significant. Data were documented in Excel v. 16.16.2 (Microsoft, USA) and analyzed with SPSS v. 30.0.0 (IBM, USA).

## Results

### Participant demographics and baseline characteristics

Baseline demographics were compared between patients with available RTW data and those excluded for missing RTW data: There were no differences in age (28.15 years (SD 7.54) vs 28.44 years (SD 8.22); p = 0.800), BMI (24.31 kg/m^2^ (SD 4.71) vs 24.31 kg/m² (SD 4.27); p = 0.994), or sex distribution (female: 85.5% vs 82.5%; p =0.574), suggesting no relevant demographic selection bias. Radiological evaluation revealed statistically significant changes in all measured parameters following PAO, including lateral centre-edge angle (p < 0.001), Tönnis angle (p < 0.001), anterior wall index (p < 0.001), posterior wall index (p < 0.001), and femoral head extrusion index (p < 0.001).

A detailed description of pre- and postoperative radiological data is shown in Table II. Functional outcome measures also improved significantly. Statistically significant differences were observed for pain (NRS, p < 0.001), hip-specific function (iHOT-12, p < 0.001), SHV, p < 0.001), and all sub-domains of the HOOS score: Symptoms (p < 0.001), Pain (p < 0.001), Function in daily living (p < 0.001), and QoL (p < 0.001). During the follow-up period, two patients (1.7%) underwent conversion to total hip arthroplasty. Both patients had sedentary preoperative occupations (REFA 0) and neither reported occupational change. No additional reoperations influencing occupational status were observed.

**Table II.** Pre- and postoperative radiological and clinical outcome parameters.

Variable	Mean preoperative (SD)	Mean postoperative (SD)	p-value
Lateral centre-edge angle, °	16.13 (6.05)	29.20 (5.76)	< 0.001
Tönnis angle, °	13.14 (6.63)	1.40 (7.27)	< 0.001
Anterior wall index	0.378 (0.153)	0.461 (0.146)	< 0.001
Posterior wall index	0.829 (0.190)	0.738 (0.250)	< 0.001
Femoral head extrusion index, %	23.85 (8.25)	9.80 (8.54)	< 0.001
NRS Pain	7.23 (1.85)	2.28 (2.10)	< 0.001
iHOT-12	40.34 (22.12)	71.33 (23.70)	< 0.001
SHV	42.18 (24.30)	79.69 (17.99)	< 0.001
HOOS Symptoms	50.81 (26.77)	71.45 (19.03)	< 0.001
HOOS Pain	42.74 (23.47)	78.71 (19.83)	< 0.001
HOOS ADL	54.52 (28.94)	83.24 (19.00)	< 0.001
HOOS QoL	28.79 (23.34)	60.58 (25.62)	< 0.001

ADL, activities of daily living; HOOS, Hip disability and Osteoarthritis Outcome Score with sub-scales for Symptoms, Pain; iHOT-12, International Hip Outcome Tool (12-item version); NRS, numeric rating scale for pain; QoL, quality of life; SHV, Subjective Hip Value.

### Comparative analysis: returnees compard with non-returnees

Among the 90 working patients included in the analysis, 58 (64.4%) returned to their preoperative job (returnees), while 32 (35.6%) reported a change in occupation after periacetabular osteotomy (non-returnees). At final follow-up, no patient was unemployed. There were no significant differences in age (30.41 years (SD 6.54) vs 30.37 years (SD 7.63), p = 0.946) or BMI (24.84 kg/m² (SD 4.43) vs 25.41 kg/m² (SD 5.51), p = 0.835) between the two groups. Significant differences were observed in several preoperative PROMs. Patients who returned to work reported lower preoperative pain levels (NRS pain: 6.97 (SD 1.80) vs 7.91 (SD 1.45), p = 0.027), higher hip-related QoL and function (iHOT-12: 43.80 (SD 22.49) vs 34.56 (SD 19.73), p = 0.093), and more favourable HOOS sub-scores. In particular, returnees had better values in HOOS Symptoms (55.60 (SD 24.69) vs 42.34 (SD 27.12), p = 0.038), HOOS Pain (47.46 (SD 20.38) vs 37.50 (SD 26.88), p = 0.087), and HOOS ADL (60.45 (SD 26.43) vs 44.05 (SD 31.09), p = 0.015). Postoperative PROMs improved in both groups, but no statistically significant differences were observed in postoperative scores for NRS, iHOT-12, SHV, or HOOS sub-scales (all p > 0.5). PASS achievement did not differ significantly between returnees (68.2%) and non-returnees (75.0%; p = 0.476). Time to RTW differed significantly with longer work absences in in non-returnees (p = 0.024). The classification of preoperative physical workload according to the REFA system differed markedly between groups. Returnees had significantly lower preoperative workload compared with non-returnees (median 0 (IQR 0 to 1) vs 2 (IQR 1 to 3), p < 0.001). Among non-returnees, postoperative REFA classification demonstrated a shift towards lower workload levels (median 0 (IQR 0 to 1)), with 71.9% classified as REFA 0 at follow-up. The duration of work absence was shorter in returnees (26.7 weeks (SD 34.0)) compared with non-returnees (33.4 weeks (SD 29.7), p = 0.024). A detailed overview of demographic details, PROMs and work-related parameters is presented in Table III.

**Table III.** Comparison of demographic, clinical, and work-related characteristics between patients who returned to work and those who did not.

Parameter	Mean returnees (n = 58) (SD)	Mean non-returnees (n = 32) (SD)	p-value
Age, yrs	30.41 (6.54)	30.37 (7.63)	0.946
BMI, kg/m²	24.84 (4.43)	25.41 (5.51)	0.835
NRS pain (preop)	6.97 (1.80)	7.91 (1.45)	0.027
NRS pain (postop)	2.07 (1.87)	2.88 (2.34)	0.077
Δ NRS	4.90 (2.32)	5.03 (2.27)	0.791
iHOT-12 (preop)	43.80 (22.49)	34.56 (19.73)	0.093
iHOT-12 (postop)	70.0 (23.1)	72.5 (26.2)	0.628
Δ iHOT-12	26.1 (31.0)	37.9 (27.7)	0.075
SHV (preop)	43.9 (24.1)	34.4 (22.6)	0.082
SHV (postop)	78.5 (17.0)	79.1 (20.5)	0.887
Δ SHV	32.8 (26.8)	43.0 (30.7)	0.105
HOOS Symptoms (preop)	55.60 (24.69)	42.34 (27.12)	0.038
HOOS Symptoms (postop)	70.7 (18.7)	69.2 (21.9)	0.738
Δ HOOS Symptoms	15.1 (28.5)	26.9 (24.5)	0.052
HOOS Pain (preop)	47.46 (20.38)	37.50 (26.88)	0.087
HOOS Pain (postop)	78.7 (20.0)	76.3 (22.0)	0.601
Δ HOOS Pain	31.3 (23.7)	38.8 (23.3)	0.151
HOOS ADL (preop)	60.45 (26.43)	44.05 (31.09)	0.015
HOOS ADL (postop)	83.7 (19.0)	78.7 (22.1)	0.267
Δ HOOS ADL	22.7 (25.3)	34.7 (26.2)	0.037
Median REFA classification (preop) (IQR)	0 (0 to 1)	2 (1 to 3)	< 0.001
Median REFA classification (postop) (IQR)	N/A	0 (0 to 1)	
Time to return to work, wks	26.7 (34.0)	33.4 (29.7)	0.024

HOOS, Hip disability and Osteoarthritis Outcome Score with sub-scales for Symptoms, Pain, and Activities of Daily Living; iHOT-12, International Hip Outcome Tool (12-item version); N/A, not applicable; NRS, numeric rating scale for pain; REFA, German workplace physical demand classification system; SHV, Subjective Hip Value.

### Reasons and timing of occupational change

Among the 32 non-returnees, 29 provided information on whether PAO was the reason for their occupational change, while three patients did not respond. Of these, 14 patients (48.3%) stated that PAO was the reason for their job change, whereas 15 (51.7%) reported that it was not. This corresponds to 15.6% of the total cohort of 90 employed patients reporting a PAO-related job change. Free-text responses indicated that persistent pain was the most frequently cited reason (five patients, 17.2%), followed by physical overload or inability to tolerate the previous workload (four patients, 13.8%), and difficulties with specific occupational tasks such as prolonged sitting or standing (three patients, 10.3%). Isolated responses included changes related to employment in gastronomy, medical foot care, or a shift towards office-based work (three patients, 10.3%). The time to job change varied widely, with a median of 86 weeks (IQR 10 to 208) after PAO. Most changes occurred between 52 and 104 weeks, while a small number of patients reported immediate changes (n = 1) or had not yet changed their occupation at the time of follow-up (n = 1).

### Change in occupational load classification among non-returnees

**Fig. 2:**
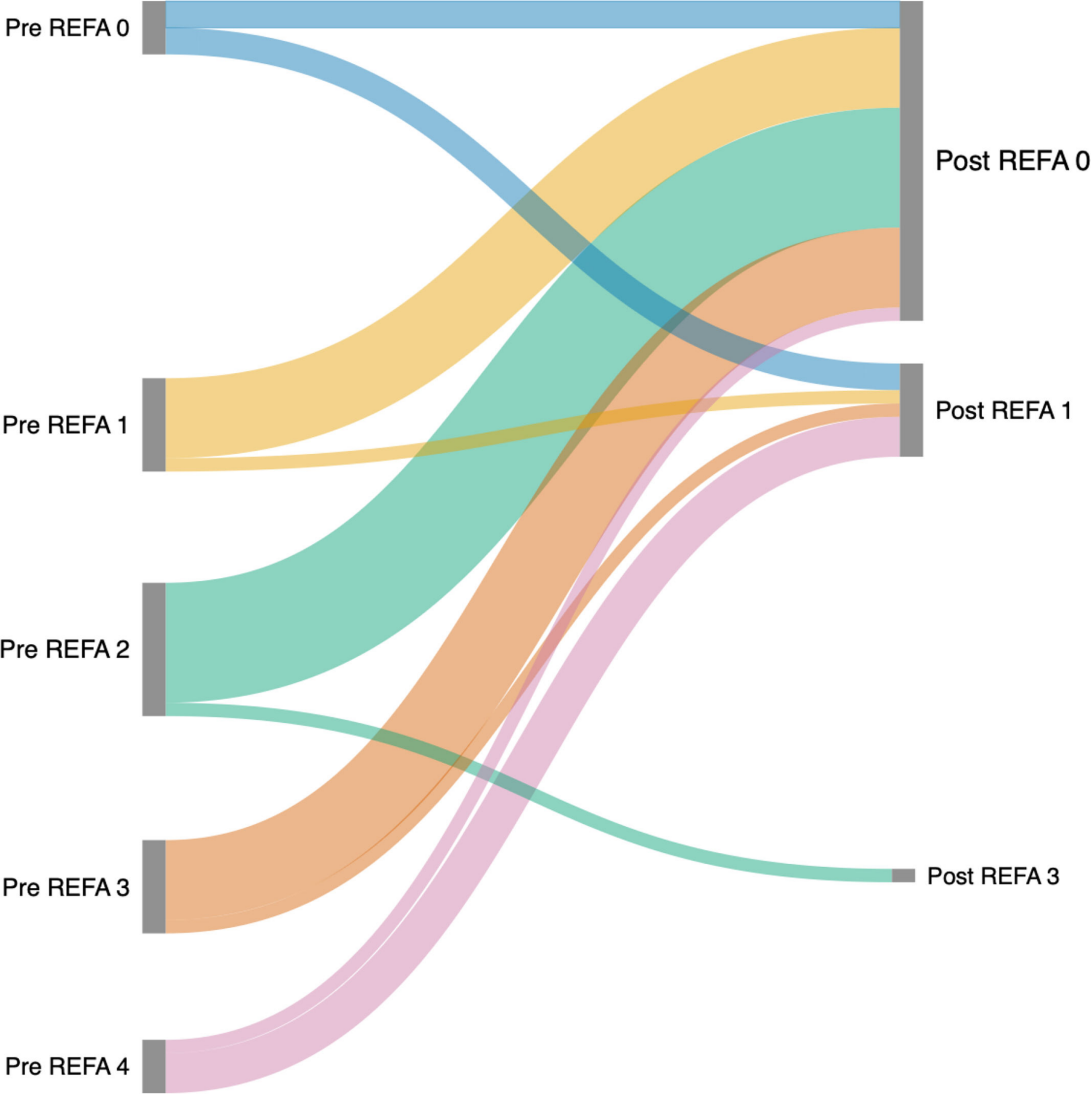
Sankey diagram illustrating the flow of non-returnees (n = 32) from preoperative to postoperative Reich Committee for Working Time Determination (REFA) classifications. Each band represents the number of patients transitioning from a specific preoperative REFA category (0 to 4, left) to their postoperative category (0 to 4, right).

Among the 32 patients who did not return to their preoperative job (non-returnees), a marked shift in occupational workload was observed based on the REFA classification system (grades 0 to 4). Preoperatively, the distribution was relatively broad: 12.5% were in sedentary jobs (REFA 0), 25.0% in light manual jobs (REFA 1), 28.1% in moderate manual jobs (REFA 2), 25.0% in heavy manual jobs (REFA 3), and 9.4% in very heavy manual jobs (REFA 4). Postoperatively, a substantial redistribution towards less physically demanding occupations was noted. The majority of non-returnees (71.9%) transitioned into sedentary work (REFA 0), while 18.8% worked in REFA 1 and only 9.4% remained in REFA 3. Notably, no patients continued in REFA 4 roles after surgery. In summary, 23 of 32 patients (71.9%) downgraded to REFA 0, suggesting a clinically relevant workload reduction in most non-returnees (Table IV). A detailed flow of REFA transitions is illustrated in the Sankey plot (Figure 2).

**Table IV.** Changes in occupational workload among non-returnees based on the Reich Committee for Working Time Determination classification system (grades 0 to 4), illustrating a postoperative shift towards less physically demanding job categories.

REFA grade	Preoperative, n (%)	Postoperative, n (%)
0 (Sedentary)	4 (12.5)	23 (71.9)
1 (Light)	8 (25.0)	6 (18.8)
2 (Moderate)	9 (28.1)	0 (0.0)
3 (Heavy)	8 (25.0)	3 (9.4)
4 (Very heavy)	3 (9.4)	0 (0.0)

REFA, Reich Committee for Working Time Determination.

### Predictors of occupational change following PAO

The regression model was statistically significant (χ² = 29.017, degrees of freedom = 10, p = 0.001) and demonstrated good overall model fit (Hosmer-Lemeshow, p = 0.610). The Nagelkerke R² was 0.379, indicating that approximately 38% of the variance in postoperative occupational change could be explained by the included predictors. The overall classification accuracy was 74.4%, correctly identifying 82.8% of patients who returned to their preoperative job and 59.4% of those who changed occupation. Among the predictors, only the preoperative REFA classification emerged as a statistically significant factor. Compared with patients with sedentary preoperative occupations (REFA 0), those in more physically demanding roles had higher odds of changing their job postoperatively. Specifically, working in REFA 1 was associated with an odds ratio (OR) of 9.97 (p = 0.005), REFA 2 with an OR of 13.11 (p = 0.001), and REFA 3 with an OR of 4.65 (p = 0.047). No interpretable results could be derived for REFA 4 due to the small number of cases in that category. In a sensitivity analysis restricted to hip-related occupational changes (n = 14), higher preoperative workload remained significantly associated with occupational change. Univariable logistic regression demonstrated that each one-grade increase in REFA classification was associated with a 67% increase in the odds of hip-related job change (OR 1.67; 95% CI 1.10 to 2.55; p = 0.016). These findings suggest that the association between physical workload and occupational change is not solely driven by non-hip-related job transitions.

### Occupational outcomes in the student sub-group

Among the total cohort of 117 cases, 27 (23.1%) were students at the time of surgery. From preoperatively to postoperatively students improved significantly for pain (NRS, p < 0.001), iHOT-12, (p < 0.001), SHV, (p < 0.001), and all sub-domains of the HOOS score (p < 0.001). In the student sub-group, PASS achievement for the iHOT-12 was observed 83.3% of cases. Follow-up data on occupational status were available for 16 of these patients (59.3%). At the time of follow-up, 14 of 16 students (87.5%) were employed, while two (12.5%) were not yet working. The postoperative REFA classification among employed students (n = 14) was category 0 in seven cases (50.0%), category 1 in six cases (42.9%), and category 3 in one case (7.1%). When asked whether the PAO had influenced their career choice, 15 of 16 students (93.8%) reported no influence, whereas one student (6.3%) indicated that the PAO had affected the decision. This student became a teacher and chose mathematics instead of sports as a subject.

## Discussion

This study investigated occupational outcomes after periacetabular osteotomy with a mid-term follow-up. Approximately two-thirds of patients returned to their preoperative occupation, while more than one third changed jobs. Importantly, in 14 cases — corresponding to 15.6% of all employed patients — the hip that underwent PAO itself was reported as the reason for occupational change. Higher preoperative workload was the strongest predictor for job change, and in the sub-group of students PAO had no relevant impact on long-term career choice.

In our cohort, 64% of patients resumed their original occupation with a mean absence of about six months. Importantly, in 14 cases (15.6% of all employed patients) PAO itself was reported as the reason for occupational change. This return-to-work rate is lower than previously reported. Fujita et al^14^ reported that 82% of patients had returned to work at one year, and Hayashi et al^20^ demonstrated almost complete return rates after curved PAO with improved productivity, although heavy workload negatively influenced outcomes. The discrepancy is most likely explained by differences in follow-up windows. Previous studies captured only early return within the first postoperative year, whereas our mid-term follow-up of approximately six years demonstrates that occupational adaptations frequently occur later, most often between one and two years after surgery. This interpretation is further supported by large single surgeon series from van Duren et al,^10^ showing that most patients had returned by six months but without the ability to capture subsequent job changes that emerge over time.^10^ Evidence from other orthopaedic fields suggests that healthcare and labour market contexts can also influence return-to-work outcomes, and such differences may partly contribute to the observed variation.^21-23^ Nevertheless, the longer follow-up appears to be the most likely explanation, as all previous PAO-specific RTW studies were limited to one year, whereas ours is the first to provide longer-term follow-up.

### Occupational change and workload

More than one third of patients in our study changed occupation after PAO, most often reducing physical workload. When comparing returnees and non-returnees, several significant preoperative differences were identified: non-returnees reported higher pain levels and worse HOOS Symptoms and ADL scores, and showed higher preoperative workload as per REFA grades. They also experienced significantly longer periods of postoperative work incapacity. These findings indicate that both subjective symptom burden and physical job demands contribute to occupational change.

When analyzing the reasons for occupational change, approximately half of the non-returnees explicitly stated that the hip that had undergone PAO, was the reason for changing their job. Persistent pain was the most frequently cited factor, followed by physical overload and difficulties with specific occupational tasks. However, this attribution should be interpreted with caution. Patients underwent PAO because of relevant preoperative symptoms and functional limitations, and the inability to fully resume preoperative occupational demands cannot be unequivocally ascribed to the surgical intervention itself. Rather, it may reflect residual limitations related to the underlying disease that are not fully reversible despite successful joint-preserving surgery. Importantly, the majority of job changes occurred not in the immediate postoperative phase but between one and two years after surgery, with some adjustments even later. This finding highlights the dynamic nature of occupational reintegration after PAO, where patients often attempt to resume their previous work initially but subsequently adapt their career path when persistent symptoms or workload demands become limiting.^10^ In addition, postoperative counselling and prevailing recommendations in the literature and social media often emphasize the avoidance of heavy physical activity for symptom control and in order to reduce the long-term risk of osteoarthritis progression or conversion to total hip arthroplasty.^3,24,25^ Such guidance, particularly when reinforced by treating physicians, may substantially influence occupational decisions independent of actual postoperative hip function. Importantly, achievement of a patient acceptable symptom state was not associated with occupational outcome. PASS rates were comparable between patients who returned to their preoperative job and those who changed occupation, indicating that job adaptations frequently occurred despite a symptom state that patients themselves considered satisfactory.

In multivariable logistic regression, only preoperative REFA classification remained an independent predictor of occupational change. Patients with heavy workload had an up to 13-fold higher risk of not returning to their original job. This observation is consistent with Hayashi et al,^20^ who demonstrated that preoperative REFA and Tönnis grades were significant predictors of reduced work productivity after curved PAO. In contrast, Fujita et al^14^ did not identify workload as a predictor, which might be due to their shorter follow-up and limited statistical power. Taken together, our data suggest that high physical workload may be a key determinant of mid-term occupational change after PAO. However, the association between REFA classification and occupational change may not solely reflect individual physical capacity. Higher REFA categories often correspond to occupations with limited opportunities for task modification or graded return-to-work options. While patients in sedentary or light-duty roles (REFA 0 to 1) may be able to adjust workload within the same job, physically demanding occupations (REFA 3 to 4) frequently lack comparable ‘light duty’ equivalents. In such settings, structural labour market constraints rather than persistent hip-related impairment alone may necessitate a change of occupation. Thus, REFA classification may partly capture not only physical workload but also the availability of workplace flexibility and modification options. This is in line with findings from other orthopaedic procedures, where preoperative workload has also been shown to influence return-to-work outcomes. After hip or knee arthroplasty, patients with lower physical workload and higher professional status returned to work earlier.^21^ Notably, this finding contrasts with the consistent and clinically meaningful improvement observed across all functional outcome measures. The apparent discrepancy between improved hip-specific function and a lack of increased occupational workload represents a central finding of the present study. It suggests that decisions regarding occupational adaptation after PAO are not driven by pain or function alone, but are substantially influenced by additional factors such as risk perception, medical advice, and broader psychosocial or occupational considerations. In this context, PAO may successfully improve hip function without necessarily restoring full preoperative work capacity, particularly in patients with physically demanding jobs. The absence of an association between PASS achievement and occupational change further supports the notion that postoperative work adaptations are not primarily driven by insufficient symptom relief. However, it cannot be excluded that comparable PASS rates between groups may partly reflect workload reduction in non-returnees, potentially leading to lower symptom provocation in daily activities. Thus, occupational adaptation itself may contribute to perceived symptom acceptability. This interplay between functional capacity and activity modification underscores the multifactorial nature of return-to-work decisions after PAO. Furthermore, these findings may suggest that occupational decisions after PAO are influenced by factors beyond hip-related pain and function, including counselling strategies, perceived long-term risks, and individual prioritization of joint preservation. However, the absence of a statistically significant difference in PASS rates should be interpreted cautiously, as comparable outcomes may partly reflect reduced occupational activity among non-returnees or limited statistical power to detect subtle between-group differences.

### Student sub-group

To the best of our knowledge, this is the first study to report occupational outcomes in students undergoing PAO. In the sub-group of students, almost all participants successfully entered the workforce after surgery. At follow-up, 14 out of 16 were employed, and only one reported that PAO had influenced career choice, by avoiding a profession with heavy physical demands. Postoperative REFA grades were predominantly sedentary or light, reflecting the shift towards occupations with lower physical workload. These findings indicate that PAO does not substantially alter long-term career trajectories for patients who undergo surgery during their training. However, as follow-up was available for only 16 of 27 students, the influence of PAO on career planning must be interpreted with caution. Given the relevance of these dynamics, further studies with larger student cohorts are warranted.

Several limitations should be acknowledged. First, the retrospective design carries an inherent risk of recall and reporting bias, particularly as work absence was self-reported. Second, our results are embedded in the specific context of the Central European labour market and social insurance system, which may limit generalizability to countries with different occupational structures and reintegration programs. Third, in the sub-group of students, only 16 of 27 could be reached for follow-up, introducing a potential non-response bias. Fourth, all patients were treated at a single high-volume centre, which may restrict external validity across different healthcare systems. Fifth, a relatively high proportion of patients lacked complete job data, which resulted in exclusion from the final analysis and may limit generalizability. However, baseline demographic characteristics did not differ between patients with and without available job information, arguing against a systematic selection bias. Finally, the relative influence of the surgical intervention itself, the pre-existing hip pathology, postoperative medical recommendations, and patient-driven risk perception on occupational outcomes cannot be disentangled. The lack of standardized assessment of postoperative counselling or information sources further limits causal interpretation.

In conclusion, PAO enables the majority of young adults with symptomatic DDH to RTW, but more than one third adapt their occupation in the mid-term. Higher preoperative workload was the dominant risk factor for occupational change, while patient-reported symptoms and function were less predictive. Importantly, occupational changes occurred most frequently beyond the first postoperative year, underlining the need for mid- to long-term follow-up when evaluating occupational outcomes. Although hip-specific function improved consistently after PAO, this did not translate into increased occupational workload, particularly in patients with physically demanding jobs. This discrepancy suggests that occupational adaptations are not solely driven by residual pain or impaired function, but are substantially influenced by factors independent of the surgical outcome, including pre-existing disease-related limitations, postoperative medical advice, and individual risk perception. In students, PAO did not meaningfully affect long-term career choice. These findings highlight the importance of nuanced preoperative counselling, particularly for patients with physically demanding occupations, and support PAO as an effective joint-preserving procedure that improves function while not necessarily restoring full preoperative work capacity in all patients.

**Take home message**

- Periacetabular osteotomy reliably improved hip-specific outcomes and enabled return to work in most patients.

- However, occupational trajectory was driven primarily by physical job demands rather than postoperative symptom relief.

## Data Availability

The data that support the findings for this study are available to other researchers from the corresponding author upon reasonable request.
